# Experimental Diabetes Mellitus in Different Animal Models

**DOI:** 10.1155/2016/9051426

**Published:** 2016-08-09

**Authors:** Amin Al-awar, Krisztina Kupai, Médea Veszelka, Gergő Szűcs, Zouhair Attieh, Zsolt Murlasits, Szilvia Török, Anikó Pósa, Csaba Varga

**Affiliations:** ^1^Department of Physiology, Anatomy and Neuroscience, Faculty of Science and Informatics, University of Szeged, Kozep Fasor 52, 6726 Szeged, Hungary; ^2^Department of Laboratory Science and Technology, Faculty of Health Sciences, American University of Science and Technology, Alfred Naccache Avenue, Beirut 1100, Lebanon; ^3^Sport Science Program, Qatar University, Doha, Qatar

## Abstract

Animal models have historically played a critical role in the exploration and characterization of disease pathophysiology and target identification and in the evaluation of novel therapeutic agents and treatments in vivo. Diabetes mellitus disease, commonly known as diabetes, is a group of metabolic disorders characterized by high blood glucose levels for a prolonged time. To avoid late complications of diabetes and related costs, primary prevention and early treatment are therefore necessary. Due to its chronic symptoms, new treatment strategies need to be developed, because of the limited effectiveness of the current therapies. We overviewed the pathophysiological features of diabetes in relation to its complications in type 1 and type 2 mice along with rat models, including Zucker Diabetic Fatty (ZDF) rats, BB rats, LEW 1AR1/-iddm rats, Goto-Kakizaki rats, chemically induced diabetic models, and Nonobese Diabetic mouse, and Akita mice model. The advantages and disadvantages that these models comprise were also addressed in this review. This paper briefly reviews the wide pathophysiological and molecular mechanisms associated with type 1 and type 2 diabetes, particularly focusing on the challenges associated with the evaluation and predictive validation of these models as ideal animal models for preclinical assessments and discovering new drugs and therapeutic agents for translational application in humans.

## 1. Background

**Figure 1:**
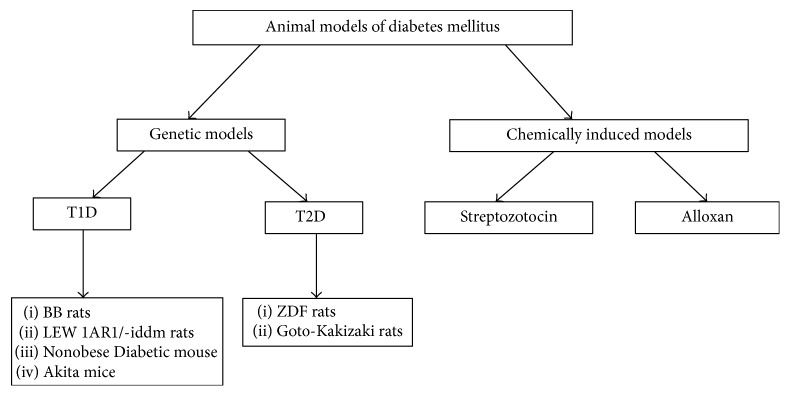
Summary of animal models of diabetes mellitus.

Diabetes mellitus is well known as a chronic metabolic disease that is characterized by a relative or absolute lack of insulin, resulting in hyperglycemia. A variety of complications arises from chronic hyperglycemia such as neuropathy, nephropathy, and retinopathy and increased risk of cardiovascular disease [1]. The two most common types of diabetes mellitus are type 1 diabetes (T1D) and type 2 diabetes (T2D). Type 1 diabetes is generally thought to be precipitated by an immune-associated, if not directly immune-mediated, destruction of insulin-producing pancreatic *β* cells [2, 3]. Therefore, it is considered as an autoimmune disease, and its occurrence is most common in children and young adults [4]. The management of the disease via blood glucose monitoring and exogenous insulin administration is arduous and costly, which in parallel with the meticulous efforts to regulate blood glucose can result in hyper- and hypoglycemic events associated with systemic comorbidities [5, 6]. Type 2 diabetes (T2D) is associated with insulin resistance and a lack of adequate compensation by the beta cells which lead to a relative insulin deficiency [7]. Therefore, both types of endocrine disorders represent quite complex diseases with the involvement of different bodily systems. On this basis, animal models should be carefully selected for diabetes investigations depending on what aspects of the disease are being studied. Furthermore, animal models play a vital role in the understanding of diabetes pathogenesis as they allow the combination of genetic and functional characterization of the syndrome [8]. Attaining deficiency in insulin production in type 1 diabetes mellitus can occur by a variety of different mechanisms ranging from chemical ablation of the beta cells to breeding rodents that spontaneously develop autoimmune diabetes. Since the animals used to study type 1 diabetes are highly inbred with a limited number of pathways to T1D, the relevance to human T1D has been questioned [9]. In type 2 diabetes (T2D) mellitus, numerous animal models have been developed for understanding the pathophysiology of diabetes and its complications [10]. These animal models tend to include models of insulin resistance and/or models of beta cell failure. On the other hand, several T2D animal models are obese, reflecting the human condition where obesity is closely linked to T2D development. Most of these models tend to have abnormalities in a single gene or multiple genes related to obesity, glucose intolerance, and/or insulin resistance leading to high blood glucose levels [11]. The outgrowth and progression of diabetic complications are affected by various factors including obesity, insulin resistance, hyperglycemia, and hyperlipidemia [10] (Figure 1).

## 2. Zucker Diabetic Fatty (ZDF) Rats

The discovery of this type of rats occurred in 1961 after a cross of* Merck (M-strain)* and* Sherman *rats. They are characterized by a mutated leptin receptor that induces hyperphagia and the rats become obese by 4 weeks of age [12]. These rats are hyperinsulinemic, hyperlipidemic, and hypertensive as well and show impaired glucose tolerance [13]. The homozygous mutation (fa/fa) of the leptin hormone receptor results in the development of type 2 diabetes in male rats when they are fed a high-energy rodent diet. These rats develop advanced insulin resistance and glucose intolerance between 3 and 8 weeks of age and turn overtly diabetic between 8 and 10 weeks of age with glucose levels in the feeding state typically 500 mg/dL by 10 to 11 weeks of age. Evidence suggests that there is a good consistency between the increase in islet DNA content and serum insulin levels indicating that islet hyperplasia plays a role in the development of hyperinsulinemia in* Zucker Diabetic Fatty (ZDF) rats* [14].

Triglycerides and cholesterol levels in obese rats are higher than those observed in lean rats. The lipotoxicity is attributed to the products of excessive non-*β*-oxidative metabolism of fatty acid excess in skeletal muscle and pancreatic islets [15–17]. The common complications of obesity, insulin resistance, cardiovascular disease, and diabetes are believed to be caused by high levels of these metabolic products through disrupting cell function and ultimately by promoting programmed cell death “lipoapoptosis” [16, 17]. In addition, it was reported that very high lipid levels can be induced in obese* Zucker Diabetic Fatty (ZDF)* rats, using high saturated fat and sucrose-containing diets. The infertility of obese males is considered an important issue that hampers research in these rats and which has been addressed by the use of testosterone propionate (TP) [18]. Depending on the amount and duration of TP administration, male obesity increases the probability of ejaculation and sexual activity. Furthermore,* ZDF rats *do not develop hypertension or cardiovascular disease spontaneously [19].

The inbred* Zucker Diabetic Fatty (ZDF) rats *substrain with a diabetogenic phenotype is derived by the induction of a mutation in this strain. This derived strain of rats are less obese than the* Zucker Fatty (ZF) rats* but have more severe insulin resistance which they are unable to compensate for due to the increase in apoptosis levels in beta cells [20]. This is characterized by initial hyperinsulinemia at eight weeks of age followed by decreased insulin levels [21]. It was mentioned that females do not develop overt diabetes [13]. According to these facts, the evolution of diabetes in male leptin receptor-deficient* ZDF rats (ZDF/CrlCrlj)* has become a popular model for preclinical studies of type 2 diabetes due to the fact that these rats exhibit disrupted islet architecture, B cell degranulation, and increased B cell death [19].

## 3. BB Rats

This type of rat was derived from outbred* Wistar rats*. Firstly, spontaneous autoimmune diabetes was identified in 1974 in a Canadian colony and then led to the creation of two founder colonies from which all substrains have been derived including one of inbred* Biobreeding Diabetes-Prone/Worcester (BBDP/Wor)* and one of outbred* Biobreeding Diabetes-Prone (BBDP) rats* [22]. It was reported that immunologically and genetically distinct* BB rat* substrains were derived from several tertiary* Biobreeding (BB) rat* colonies [23] including* Biobreeding/Ottawa Karlsburg (BB/OK) (BB/Pfd)* and* Biobreeding Spontaneously Hypertensive Rats (BB.SHR)* [24–27]. Additionally,* BB rats* resistant to diabetes have been bred to act as controls.

After puberty,* Biobreeding (BB) rats* develop diabetes with a similar incidence between males and females with about 90% of rats developing diabetes between eight and sixteen weeks of age. The diabetic phenotype is quite severe and is characterized by development of hyperglycemia, hypoinsulinemia weight loss, and ketonuria requiring insulin therapy for survival. The latter clinical and metabolic symptoms are preceded by histological abnormalities in the islets of pancreas. It was concluded that the earliest detectable anomaly is the enhanced expression of interferon-*α* (IFN-*α*) and major histocompatibility complex (MHC) class I molecules in islet cells, which occur soon after weaning followed by progressive infiltration of islets by macrophages, natural killer (NK) cells, dendritic cells, T cells and to a lesser extent B cells [28–30].

Though these animals have insulitis with the presence of T cells, B cells, macrophages, and natural killer (NK) cells, they are lymphopenic with a severe reduction in CD4^+^ T cells and a near absence of CD8^+^ T cells. Lymphopenia is not a characteristic of type 1 diabetes (T1D) neither in humans nor in* Nonobese Diabetic (NOD) mice*. Recently, a novel member of the GTPase family was described as rIAN5 in the BB rat, and its mutation, which was identified in the Gimap5 (Ian5) gene (RNO4), results in T cell lymphopenia causing diabetes in this model [31, 32]. Consequently, it is perceived as a disadvantage in using Biobreeding* (BB) rats* as a model of human type 1 diabetes [22]. T lymphopenia is present at birth and its severity explains why this animal is immune-deficient. Although the serum of* BB rats* contains normal levels of circulating immunoglobulins and autoantibodies of various specificities, their capability to generate germinal centers following immunization with T-dependent antigens is compromised and the ability of T cells to generate allospecific cytotoxic T cells and to proliferate robustly in response to alloantigens in vitro is severely reduced [33–36]. Otherwise, the advantages of this model have been insufficient in elucidating type 1 diabetes (T1D) on the genetic level [37]. In some intervention studies and studies of diabetic neuropathy,* BB rats* have been used as well [38–40]. Both sexes of inbred* Biobreeding Diabetes-Prone/Worcester (BBDP/Wor) rats* and outbred* Biobreeding Diabetes-Prone (BBDP) rats* develop pancreatic insulitis that is rapidly followed by selective destruction of beta cells and frank diabetes between 50 and 90 days of age. The natural course of insulitis in the spontaneously diabetic BB rat was noted to be different from that of the* Nonobese Diabetic (NOD) mouse*. In* Biobreeding (BB) rats*, insulitis is morphologically similar to what is observed in human T1D and features a predominance of Th1-type lymphocytes [41, 42]. Profound T cell lymphopenia is shown clearly to be the most obvious and problematic immunopathology in all diabetic* Biobreeding (BB) rats* [43–45]. The rats also appear to be severely deficient in ART2^+^ T cells, where ART2 is a rat maturational T cell alloantigen with nicotinamide adenine dinucleotide glycol-hydrolase activity that appears to identify cells with immune-regulatory properties [46]. The conditions that lead to beta cell autoreactivity in the spontaneously diabetic* BB rat* are still not resolved yet, but the mechanism likely involves the presentation of autoantigen by* RT1* ^*u*^ molecules, while the identity of the primary autoantigen is unknown. The transfusion of CD4^+^ ART2^+^ T cells to overcome the effects of lymphopenia is considered to be one of the unique preventative strategies against the spontaneous diabetic* BB rat* model [47] with no documented human equivalent of the ART2^+^ T cell. The treatment of diabetic* BB rats* has been achieved with islet transplantation plus either immunosuppression [48] or costimulatory blockade [49].

## 4. LEW 1AR1/-iddm Rats

### 4.1. (ZDF-Lepr^fa^/Crl)

This rat model of type 1 diabetes (T1D) arose spontaneously in a colony of congenic Lewis rats characterized by a defined MHC haplotype* Lewis.1AR1 (LEW.1AR1) rat. *It is a unique model for studying human T1D in which rats were being bred in Hannover Medical School (Ztm) at the Institute of Laboratory Animal Science and obtained as a result of a spontaneous mutation in the* LEW.1AR1* strain. It was obvious that these rats develop diabetes between 60 and 90 days of age and are characterized by rapid progression of insulitis leading to extensive *β* cell destruction [50, 51]. These rats are distinct from the well-characterized* Nonobese Diabetic (NOD)* model of T1D as, unlike* Biobreeding Diabetes-Prone (BBDP) rats*, diabetes develops with equal frequency in both male and female animals [52]. Studies clearly showed that the incidence of diabetes in these animals is increased from 20% to 60% with further inbreeding with equal incidence in both genders [50, 51]; moreover, the animals also display a prediabetic state that lasts for approximately a week with islet infiltration [51]. The* Lewis-insulin dependent diabetes mellitus (LEW-iddm) rats* are able to survive well after diabetes is overtly shown and, consequently, they can be used as a model to study diabetic complications [53]. These animals are ketonuric, but they are not lymphopenic expressing normal numbers of ART2^+^ T cells. Furthermore, *β* cells of the islets of Langerhans of affected animals are infiltrated with B and T lymphocytes, macrophages, and natural killer (NK) cells and it appears that the beta cells die via apoptosis [22]. They show apoptotic beta cell destruction induced by proinflammatory cytokines that are released from islet-infiltrating immune cells [51]. In* Lewis.1AR1-insulin dependent diabetes mellitus (LEW.1AR1-iddm) rat*, the diabetic syndrome displays an autosomal recessive mode of inheritance with an incomplete penetrance of the mutant phenotype of about 60% [50, 51]. The genome-wide linkage analysis using a* (BN6LEW.1AR1-iddm) × LEW.1AR1-iddm] N2 (N2 BN)* population facilitated the discovery of three T1DM susceptibility loci in this rat model [54]. One of these loci mapped to RNO20p12 within the MHC region provides T1D mellitus susceptibility also in humans (*IDDM1*) and the* NOD mouse (idd1)* and the* BB rat* and the* Komeda diabetes-prone* (*KDP) rat* models* insulin dependent diabetes mellitus1 (Iddm1)* [8]. In this case, the MHC haplotype plays a pivotal role in permitting T1DM development [55]. It was possible to confer protection against diabetes through adoptive transfer of immune cells from the* Lewis.1AR1 (LEW.1AR1)* background strain into prediabetic* LEW.1AR1*-*iddm* rats. Thus,* LEW.1AR1* rats are protected against beta cell autoreactive immune cells by the regulatory elements of the immune system [56]. In addition to the diabetes-related MHC class II (*Iddm1*) susceptibility locus,* insulin dependent diabetes mellitus8 (Iddm8)* was identified recently as the site of mutation(s) contributing to disease susceptibility in the* LEW.1AR1 strain* [57].

Moreover, in addition to autoimmune diabetes,* Lewis.1AR1-insulin dependent diabetes mellitus (LEW.1AR1-iddm)* rats manifest a second phenotype described as a “variable CD3^+^ T cell frequency” which could also be mapped within the Iddm8 region [8]. So, the mutation in the Iddm8 region confers susceptibility to diabetes as well as to the variable CD3^+^ T cell frequency in blood. A previous study done by Arndt et al. shows that the spontaneous mutation in the LEW.1AR1-iddm rat was also identified in the* Dock8* gene, and this mutation was also shown to be accompanied by autoimmune diabetes development and a variable CD3^+^ T cell frequency in peripheral blood. It is well known that other mutations in the Dock family can also lead to an imbalance in the immune system in humans [58–60].

Effective prevention strategies from diabetes require a combination therapy to target the proinflammatory cytokines produced in the different immune cell types. Of crucial importance in this context is the proinflammatory cytokine tumor necrosis factor-*α* (TNF-*α*) that is expressed in all immune cell types infiltrating the pancreatic islets in patients with T1D as well as in the* LEW.1AR1-iddm* rat model [61, 62]. Fingolimod (FTY720) treatment has been performed to protect against islet infiltration in the prediabetic and early diabetic phase in type 1 diabetes, but only in animal models and not in humans [63, 64]. The clinical benefit of this immunomodulatory agent is the lack of severe adverse effects at a dose of 1 mg/kg body weight as applied in diabetes prevention studies in rodent models, including the* LEW.1AR1-iddm (iddm)* [65].

The* MHC-II RT1-B/D* ^*u*^ haplotype is indispensable for diabetes development in rats, and, in several studies, the congenic Lewis* (LEW), Lewsi.1AR1 (LEW.1AR1),* and* Lewsi.1WR1 (LEW.1WR1)* strains were selected to compare the frequencies of immune cell subpopulations in peripheral blood to those* in LEW.1AR1-iddm rats*. The* LEW.1AR1 (RT1-A* ^*a*^ *, RT1-B/D* ^*u*^, and* RT1-C* ^*u*^) and* LEW.1WR1 (RT1-A* ^*u*^ *, RT1-B/D* ^*u*^, and* RT1-C* ^*a*^) strains express also the* MHC-II RT1-B/D* ^*u*^ haplotype, and, conversely, the original* LEW (RT1-A* ^*l*^ *, RT1-B/D* ^*l*^, and* RT1-C* ^*l*^) strain cannot develop diabetes because of the missing* RT1-B/D* ^*u*^ haplotype.

## 5. Goto-Kakizaki Rats

Developing mild hyperglycaemia at an early stage of life, the* Goto-Kakizaki (GK)* rat is considered a nonobese model. This type 2 diabetes (T2D) model originates from* Wistar (W)* rat established by repeated inbreeding with* Wistar (W) *rats at the upper limit of normal distribution for glucose tolerance [66, 67] with most likely glucose intolerance due to impaired *β* cell mass and function on the background of a polygenic inheritance. Chronic exposure to hyperglycemia (glucotoxicity) may further impair *β* cell function and insulin action and contribute to the development of hyperglycemia. However, insulin resistance contribution may likely be secondary in the development of hyperglycemia in this model.* Goto-Kakizaki (GK) rat* pancreatic islets may develop into the so-called starfish-shaped islets characterized by disrupted structure with clear fibrosis separating strands of endocrine cells making the islets resemble the appearance of a starfish. These changes are not present in the pancreas of young* GK rats* but increase in prevalence with aging [68]. In adult* Goto-Kakizaki (GK) rats*, total pancreatic *β* cell mass and pancreatic insulin stores are similarly decreased by 60% [69, 70]. This alteration in the *β* cell population cannot be attributed to increased *β* cell apoptosis but is rather related, at least in part, to marked decrease in *β* cell replication [71]. The onset of a profound alteration in glucose-stimulated insulin secretion by the* GKβ* cell, after weaning, correlates with the exposure to the diabetic environment. These changes in the islet function may result, at least in part, from a loss of differentiation of *β* cells chronically exposed to even mild chronic hyperglycemia and elevated plasma nonesterified fatty acids, a process referred to as “glucolipotoxicity.” According to a previous study conducted in our laboratory using* GK rats* as a diabetic model, measurement of oral glucose tolerance test (OGTT) revealed that blood glucose concentration peaked at 60 min after glucose administration and then began to decrease. Basal blood glucose level was significantly lower than that after the oral glucose tolerance test (OGTT) pointing to the impairment of carbohydrate homeostasis. Furthermore, the study revealed that hyperglycemia in* GK rats* can be moderated by exercise, where exercise promotes the prevention and treatment of type 2 diabetes (T2D) because of the increase in the capacity of the muscles to capture circulating glucose due to the decreased intramuscular fat reserves. Running* Goto-Kakizaki (GK) rats* displayed a significantly lower fasting blood glucose level after the 6-week training period. Initial basal glucose level (0 min) was 9.17 ± 0.21 mM/L in the sedentary group and 6.82 ± 0.3 mM/L in the voluntary running group. Exercise was associated with a significantly lower blood glucose level after 60 (20.51 ± 0.58 versus 16.39 ± 0.81 mmol/L) and 120 (19.61 ± 0.76 versus 14.58 ± 0.88 mmol/L) min of the OGTT test [72].

## 6. Chemically Induced Diabetes

Alloxan and streptozotocin (STZ) are considered the most potent diabetogenic chemicals used in diabetes research so far [73]. Both chemicals are employed as cytotoxic glucose analogues that tend to accumulate in pancreatic beta cells through glucose transporter 2 (GLUT2) [73].

**Figure 2:**
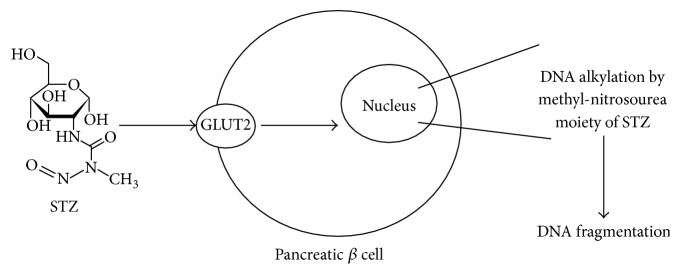
The mechanism of action of streptozotocin (STZ) in *β* cells.

STZ acts as a nitrosourea analogue, in which there is linkage between the N-methyl-N-nitrosourea (MNU) moiety and carbon-2 of hexose [73]. In general, the mode of action of toxicity of STZ depends on the DNA alkylating activity of its methyl-nitrosourea moiety [73]. The transfer of the methyl group from STZ to the DNA molecule causes damage along a defined chain of events [73] and leads to DNA fragmentation [73] (Figure 2).

STZ-induced diabetes is of two types: the adult type and neonatal type. In case of the adult type streptozotocin- (STZ-) induced diabetes, rats weighing 140 to 300 g underwent a single streptozotocin (STZ) intraperitoneal injection (45–70 mg/kg) dissolved in 0.1 M citrate buffer (pH 4.5) after an overnight fast. Control rats of the same age received only an injection of citrate buffer.

A number of studies with STZ-induced diabetes mellitus reported that diabetes mellitus can improve the recovery of cardiac function after ischemia-reperfusion [74] along with decreasing the incidence of arrhythmias [75, 76]. It was previously contended that the contractile function of diabetic hearts following ischemia-reperfusion recovered to a greater extent than that of the control hearts. However, the magnitude of the preischemic basal contractile function was significantly reduced in the diabetic hearts [74]. Protein kinase C (PKC) inhibitors were found to restore the impaired cardiac function in diabetes before ischemia; however, PKC inhibitors completely abolished protection against postischemic injury in diabetic hearts [74]. This effect of protein kinase C inhibition in diabetes may be explained by the decreased phosphorylation of myocardial proteins (e.g., troponin) [74]. Chen et al. have found that severe hyperglycemia due to diabetes mellitus induced by STZ decreases the incidence of arrhythmias and increases coronary flow and expression of heat shock protein 90 (hsp90) [75]. Nonetheless, the study hypothesized that increased osmolarity due to severe hyperglycemia is in charge of the overexpression of heat shock protein 90 (hsp90), which in turn increases the production of endothelial nitric oxide (NO) [75]. Furthermore, it was well addressed that diabetic hearts are much more resistant to ischemia-induced arrhythmias compared to nondiabetic controls [76]. It was also noted that, 4 weeks following streptozotocin (STZ) treatment, hearts have improved functional recovery after ischemia-reperfusion in comparison to control hearts and this functional recovery was associated with the prevention of hexokinase solubilization during ischemia [77].

Conversely, several published studies indicated that diabetes mellitus induced by STZ did not decrease the infarct size [78–82], with no reduction in the incidence of arrhythmias [83]. Some studies also reported that STZ-induced diabetes mellitus even increased the infarct size compared to normal rats [84–86].

## 7. Diabetes and Cardioprotective Strategies

It is still not clear whether or not preconditioning can exert a cardioprotective effect in subjects with diabetes mellitus, and further studies are needed in this regard. Wu et al. found that pharmacological preconditioning with ginsenoside Rb1 in diabetic rats decreased infarct size, while phosphoinositide 3 (PI3) kinase inhibition with wortmannin reverted the cardioprotective effects of ginsenoside Rb1, which is an indication that pharmacological preconditioning with ginsenoside Rb1 involved PI3 kinase/Akt signaling in the diabetic hearts [85]. A study by Zhu et al. showed that the infarct size is decreased in diabetic rats subjected to ischemic preconditioning and noninvasive limb ischemic preconditioning, and this protective effect involved the increase in enzymatic activity of superoxide dismutase as well as increased glutathione peroxidase activity [87]. Pharmacological preconditioning with remifentanil decreased the infarct size both in normal and in diabetic rats and this protection involves antiapoptotic pathways of survival, including extracellular signal regulated kinases (ERK); however, pharmacological preconditioning was less efficient in the case of diabetic hearts [78]. Another study by Ravingerová et al. on isolated hearts taken from rats suffering from diabetes mellitus induced by a single streptozotocin (STZ) intravenous injection (45 mg/kg) showed that ischemic preconditioning induced an antiarrhythmic effect only during the chronic stage of diabetes, in contrary to the acute stage, where ischemic preconditioning did not decrease the incidence of ischemic arrhythmia.

Several other studies also revealed that diabetes impairs preconditioning. Similarly, a study by Ji et al. proved that diabetes impaired preconditioning [81]. Ischemic preconditioning was abolished in STZ-treated rats due to a failure to increase glucose uptake during reperfusion [81]. It was shown by Vinokur et al. that impaired iron homeostasis in diabetic heart can lead to failure of preconditioning to induce myocardial protection [82]. Sharma et al. found that diabetes mellitus impaired pharmacological preconditioning by adenosine; however, adenosine transport blockade by dipyridamole restored the protective effect [86]. Results of Ajmani et al. suggested that attenuation of cardioprotection in the diabetic heart may be due to the decrease in ischemic preconditioning-mediated release of nitric oxide (NO) in the diabetic myocardium, which may be a result of upregulating caveolin and the subsequent decrease of endothelial nitrogen oxide synthase (eNOS) activity [80]. According to Yadav et al., diabetes mellitus induced attenuation of cardioprotective effect of ischemic preconditioning through the activation of glycogen synthase kinase 3-beta (GSK-3*β*), due to the impaired protective upstream signaling pathways and (mPTP) opening during reperfusion [79]. On the other hand, Tosaki et al. reported that ischemic preconditioning did not confer antiarrhythmia and antimyocardial stunning effects in diabetic rats induced by intraperitoneal injection of STZ (65 mg/kg) [83].

Ischemic postconditioning, as another strategy in cardioprotection, is abolished by STZ-induced diabetes mellitus. According to Ren et al., it was found that ischemic postconditioning failed to ameliorate the cardiac function and most likely failed to reduce creatine kinase and cardiac troponin I release in isolated perfused heart from diabetic rats. Moreover, it was also revealed in this study that the decrease in transient receptor potential cation channel subfamily V member 1 (TRPV1) and the increase in the expression of calcitonin gene related peptide (CGRP) and Substance P (SP) in diabetic hearts caused the loss of the cardioprotective activity of ischemic postconditioning associated with a failure to increase calcitonin gene related peptide (CGRP) and Substance P (SP) release [88]. Sevoflurane postconditioning was abolished by chemically induced diabetes in a rat model and this detrimental effect involved the impairment of phosphorylation of glycogen synthase kinase 3-beta (GSK-3*β*) and its upstream pathways, such as PI3 kinase/Akt and extracellular signal regulated kinases (ERK) in diabetes [89]. Najafi et al. indicated that ischemic postconditioning did not exert a protective effect in diabetic hearts [90]. Concurrent inhibition of 1-methyl-4-phenyl-1,2,3,6-tetrahydropyridine (mPTP) may lead to a restoration of the protective effect of ischemic postconditioning in these diseased hearts [90].

In case of neonatal type of STZ treatment, 2–4-day-old neonatal* Wistar (W) rats* are injected intraperitoneally with 65–100 mg/kg of STZ or its vehicle (ice-cold citrate buffer) to induce diabetes. Neonatal rats are kept with their mother during the lactation period until week 4. For lactating mothers, standard rat chow and tap water were supplied ad libitum [91]. Type 2 diabetes (T2D) induced by neonatal STZ treatment can protect the heart against ischemia-reperfusion [92]. Moreover, preconditioning can furthermore protect the diabetic heart [92]. Based on a previous study, the recovery of cardiac function after ischemia-reperfusion and infarct size was improved in diabetic rats [93].

**Figure 3:**
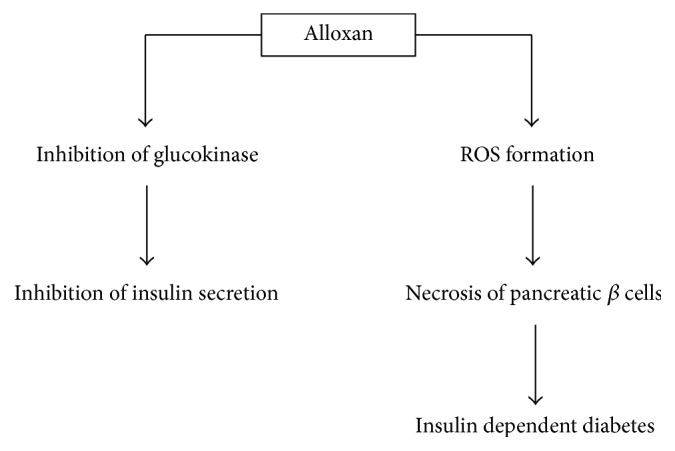
The main pathological effects of alloxan.

It is well known that alloxan possesses two pathological effects: it can selectively inhibit glucose-induced insulin secretion through the inhibition of glucokinase, the glucose sensor of the beta cell, and it creates a state of insulin dependent diabetes by inducing reactive oxygen species (ROS) formation causing a selective necrosis of beta cells [73] (Figure 3).

Alloxan was also shown to induce diabetes in fasting male* Wistar (W)* rats weighing 200–250 g that were given subcutaneously 125 mg/kg alloxan [94]. Galagudza et al. have found that diabetic rats for six weeks were more resistant to ischemic damage compared to the myocardium of healthy animals, and ischemic preconditioning in animals with diabetes mellitus was much less effective in limiting infarct size than in control animals [94]. However, diabetes mellitus induced by alloxan interferes with the protective effect of postconditioning in other species [95–97].

## 8. Nonobese Diabetic Mouse

This type of mouse was first developed at Shionogi Research Laboratories in Osaka, Japan, in 1974 [98]. Insulitis appears at around 3rd or 4th week of their age. During this prediabetic stage, the islets of the pancreas become infiltrated by CD4^+^ and CD8^+^ lymphocytes, though natural killer (NK) and B cells are also present [99]. The infiltration of innate immune cells into the islets attracts adaptive CD4^+^ and CD8^+^ T cell subsets into the islets starting from approximately 4–6 weeks of age [100], which are both required for diabetes development. Insulitis leads to the destruction of beta cells, while the onset of overt diabetes usually appears when approximately 90% of the pancreatic insulin is lost at around 10–14 weeks, although diabetes can develop up to 30 weeks of age. These mice can lose weight rapidly when they become overtly diabetic and require insulin treatment.

*Nonobese Diabetic (NOD)* mouse is one of the most commonly used models to study type 1 diabetes (T1D). Unlike other models used in autoimmunity studies, this model can develop spontaneous disease similar to humans. Several advances in our understanding of the disease arose from using this model, including the identification of several autoantigens and biomarkers that are similar in humans and enabled the development of therapeutic targets [101]. Both in* NOD* mice and in humans, the most important genetic factor that contributes to T1D susceptibility is the major histocompatibility complex (MHC) known as insulin dependent susceptibility 1 (idd1) in mice and* insulin dependent diabetes mellitus1 (IDDM1)* in humans [102, 103]. In addition, more than 40 genetic loci discovered in both* NOD* mice and humans have been shown to play an important role in mediating T1D susceptibility, including genes related to immune system function and regulation as well as pancreatic beta cell function [104]. The pancreas of* Nonobese Diabetic (NOD)* mice is infiltrated by innate immune cells at an early period of 3 weeks of age. These cells include dendritic cells (DCs), macrophages and neutrophils [105–107], prior to the infiltration of the lymphocytes. Identically, these cells are also found in the human islet infiltrate [108]. According to several studies, MHC class 2 proteins in* NOD* mice share structural similarities to those in humans, which may confer resistance or susceptibility to the disease in both* NOD mice* and humans [109]. The similarity in the genes of type 1 diabetes (T1D) between* NOD mice* and humans has been completely useful in dissecting some mechanisms and pathways behind T1D [110]. Therefore, these animals are believed to be a potentially suitable model for testing therapies in which modulation of the autoimmune response is being targeted. However, it should also be noted that there are a number of effective drugs in* NOD mice*, which were shown to be ineffective in humans [111]. One of the main issues is the time point of intervention and another difficulty in the translation of therapies tested in* Nonobese Diabetic (NOD) mice* is that whereas the pancreas of* NOD* can be removed for examination after a study, there is a deficiency of biomarkers in human peripheral blood that could be used as evidence to verify the success of the intervention [112]. There are problems as well in translating dosing from the* Nonobese Diabetic (NOD) *mouse to humans [112]. Diabetes development in the* Nonobese Diabetic (NOD)* mouse is associated negatively with microbial exposure. Therefore, mice should be protected in specific pathogen-free (SPF) conditions to maintain diabetes incidence. Because of the differences in gender, the unpredictability of the disease onset, and the requirement for SPF conditions, this mouse model is expensive to maintain as type 1 diabetes (T1D) model in comparison with the chemically induced diabetic model. Adoptive transfer was also proved to be useful, where this strategy is based on the injection of T cells from diabetic* NOD* mice into nondiabetic recipient mice, causing the recipient mouse to develop diabetes [113].

## 9. Akita Mice

**Figure 4:**
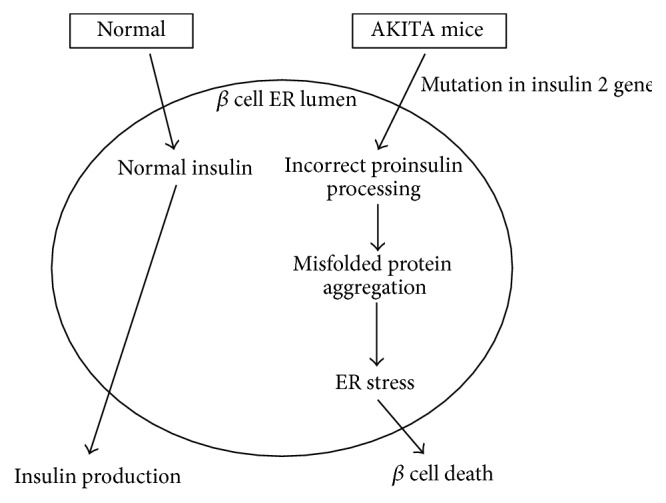
Akita mice.

The* Akita* mouse was initially developed in Akita, Japan, from a* C57BL/6NSlc* mouse due to a spontaneous mutation in* insulin 2* gene leading to incorrect proinsulin processing. This mutation caused the aggregation of misfolded proteins and led, subsequently, to endoplasmic reticulum (ER) stress (Figure 4).

Collectively, these alterations resulted in pronounced insulin dependent diabetes with an onset of 3 to 4 weeks of age. The resulting rodent model exhibits characteristic signs, including hyperglycemia, hypoinsulinemia, polyuria, and polydipsia. The absence of beta cell mass in this mouse model renders it a valid alternative to STZ-treated mice in transplantation studies. It has been well utilized as a model of type 1 diabetes (T1D) macrovascular disease and neuropathy [114–116]. Likewise, this model is usually used to investigate potential alleviators of ER stress in the islets. In this regard, some of the pathological manifestations of type 2 diabetes (T2D) are also visible in the* Akita *mouse model [117]. Studies have reported that mice and humans lacking PERK, a critical component of the ER stress response, develop spontaneous diabetes mellitus at a very young age taking into consideration that *β* cells may be especially vulnerable to endoplasmic reticulum (ER) stress [118].

*Akita* mice carry the* Ins2* ^*+*^ */C96Y* mutation, a single nucleotide substitution in the insulin 2 (Ins2) gene. This mutation causes incorrect folding of the insulin protein, pancreatic *β* cells toxic injury, and reduced insulin secretion propensity resulting in T1D [119, 120]. The* Ins2C96Y* mutation present in the* Akita *diabetic mouse prevents the formation of an essential disulfide bond between insulin 2 chains and prevents proper folding and processing of this protein. The misfolded proinsulin 2 is not secreted and is retained in the pancreatic *β* cell endoplasmic reticulum (ER) during its processing in the secretory pathway [121]. Mice carrying the* Ins2C96Y* mutation develop progressive diabetes mellitus, a phenotype that probably reflects a holistic effect rather than just the loss of correctly folded insulin production by the mutant Ins2 allele. In fact, rodents have two insulin genes (Ins1 and Ins2) and, interestingly, the loss of both Ins2 genes copies is fully compensated [122]. Even though newborn* Ins2C96Y* mutant mice have normal sized islets of Langerhans that produce an adequate complement of insulin-producing *β* cells, over time they undergo a progressive and marked loss of the *β* cell mass. Apoptosis-mediated loss of these cells correlates with the development of diabetes mellitus. A key finding was that this specific pathophysiological aspect of the* Akita* mouse can be reproduced in vitro by expressing high levels of* Ins2C96Y*, but not wild-type Ins2, in the *β* Min6 cell line [123]. This* “Akita”* polymorphism of insulin 2 gene (Ins2^akita^) acts in a dominant-negative manner in Ins2akita/^+^ mice and leads to severe hyperglycemia persisting throughout the mouse life [124]. It is likely, therefore, that the expression of* Ins2C96Y *is toxic to islet cells and that the loss of *β* cell mass mediates hyperglycemia development in the* Akita *mouse. Studies have reported that* Ins2 Akita* mouse is a valid model of diabetic sympathetic autonomic neuropathy corresponding closely to the characteristic pathology of other rodent models and humans [125].* C57BL/6-Ins2* ^*+*^ */C96Y* mice also had higher levels of albuminuria and systematic renal pathological changes. Therefore, the* C57BL/6-Ins2 Akita* model presents a number of advantages over the chemically induced STZ model of diabetes and may serve as a platform for developing models of diabetic nephropathy [126]. Nonetheless, proteinuria is less developed in* C57BL/6-Ins2* ^*+*^ */C96Y* mice and mesangial expansion is the only reported renal pathological change. In this regard,* Ins2 Akita* mice can serve as a model of diabetes ameliorating the deficiencies encountered in the STZ treatment in studies of islet transplantation [114]. It was argued as well that the mouse genetic background might exert an effect on the development of kidney injury in* Akita* mice [120]. One of the drawbacks that marred the utilization of this animal model is that the* Akita* mouse has not been validated by antineuropathic drugs [127]. These mice, for a reason yet to be identified, show mesangial immunoglobulin A (IgA) deposits [128]. This deposition is a major factor in the development of human mesangial proliferative glomerulopathy and, consequently, the lack of clear understanding of the underlying mechanism of the increase of mesangial matrix will make these observations of limited value. This is especially evident in the cases where the key endpoint is represented by alterations in mesangial volume and matrix accumulation [129].

## 10. Conclusions and Future Outstandings

Due to the increase in the prevalence of diabetes mellitus worldwide, the diabetic rat models are believed to play an important role in elucidating the pathogenesis of human diabetes and its complications, such as retinopathy, nephropathy, and neuropathy. Moreover, the diabetic rat models are essential for investigating and developing novel drugs for diabetes and its complications. All of the rat and mouse models available to date possess important limitations, despite all the advantages they offer. Thus, designing better models of diabetes mellitus (DM) that will allow identification of the underlying mechanisms or testing of therapeutic interventions, must continue. The best model of diabetes should have some major criteria such as the following: the model should have all major pathogenesis of diabetes with other minor pathogenesis that can be normally found in diabetic humans, the model should be sensitive to antidiabetic drugs, and the model needs to be suitable to study the pathogenesis of disease as well as for routine pharmacological screening of antidiabetic drugs. Furthermore, the diseased animal models have contributed to helping scientists and researchers to better understand the mechanisms of other diseases in preclinical studies that allowed the screening of drugs and pharmaceutical agents, but their value in predicting the effectiveness of treatment strategies in clinical trials has remained controversial. In this case, more future studies are needed to investigate the role of the diseases animals in combination with pharmacological therapy.
